# Self-perception of leadership styles and behaviour in primary health care

**DOI:** 10.1186/s12913-016-1819-2

**Published:** 2016-10-12

**Authors:** Glòria Jodar i Solà, Joan Gené i Badia, Pilar Delgado Hito, M. Antonia Campo Osaba, Jose Luís Del Val García

**Affiliations:** 1 Public Health Nursing, Mental, and Maternal and Infant Health, Universidad de Barcelona, Campus Bellvitge, c/Feixa Llarga s/n. 08907 L’Hospitalet de Llobregat, Barcelona, Spain; 2 Public Health Departament, Consorci d’Atenció Primària de Salut de l’Eixample (CAPSE), Barcelona University, Roselló 161, 08036 Barcelona, Spain; 3 Fundamental and Surgical-Medical Department, Universidad de Barcelona, Campus Bellvitge, c/Feixa Llarga s/n. 08907 L’Hospitalet de Llobregat, Barcelona, Spain; 4 Institut Universitari d’Investigación en Atenció Primària (IDIAP) Jordi Gol Unidad de investigación de Barcelona, Gran Via de les Corts Catalanes 587, àtic, 08007 Barcelona, Spain; 5 Barcelona’s Primary Health Care Research, Evaluation and Quality Unit Institut Català de la Salut, Numància n° 23, 08029 Barcelona, Spain

**Keywords:** Leadership, Primary health care, Self-concept, Job satisfaction, MLQ, Managers

## Abstract

**Background:**

The concept of leadership has been studied in various disciplines and from different theoretical approaches. It is a dynamic concept that evolves over time. There are few studies in our field on managers’ self-perception of their leadership style. There are no pure styles, but one or another style is generally favoured to a greater or lesser degree. In the primary health care (PHC) setting, managers’ leadership style is defined as a set of attitudes, behaviours, beliefs and values. The objectives of this study were to describe and learn about the self-perception of behaviours and leadership styles among PHC managers; to determine the influence of the leadership style on job satisfaction, efficiency, and willingness to work in a team; and to determine the relationship between transformational and transactional styles according age, gender, profession, type of manager years of management experience, and the type of organization.

**Methods:**

To describe leadership styles as perceived by PHC managers, a cross sectional study was performed using an 82 items-self-administered Multifactor Leadership Questionnaire (MLQ). This questionnaire measures leadership styles, attitudes and behaviour of managers. The items are grouped into three first order variables (transformational, transactional and laissez-faire) and ten second order variables (which discriminate leader behaviours). Additionally, the questionnaire evaluates organizational consequences such as extra-effort, efficiency and satisfaction.

**Results:**

One hundred forty responses from 258 managers of 133 PHC teams in the Barcelona Health Area (response rate: 54.26 %). Most participants were nurses (61.4 %), average age was 49 years and the gender predominantly female (75 %). Globally, managers assessed themselves as equally transactional and transformational leaders (average: 3.30 points).

Grouped by profession, nurses (28.57 % of participants) showed a higher transactional leadership style, over transformational leadership style, compared to physicians (3.38 points, *p* < 0.003). Considering gender, men obtained the lowest results in transactional style (*p* < 0.015). Both transactional and transformational styles correlate with efficiency and job satisfaction (*r* = 0.724 and *r* = 0.710, respectively).

**Conclusions:**

PHC managers’ self-perception of their leadership style was transactional, focused on the maintenance of the status quo, although there was a trend in some scores towards the transformational style, mainly among nurse managers. Both styles correlate with satisfaction and willingness to strive to work better.

## Background

The concept of leadership has been studied in various disciplines and from different theoretical approaches. It is a dynamic concept that evolves over time. For this reason, scientific literature has defined leadership in different ways. In general, it is defined as a multidimensional process, involving a position of influence within a group, in a setting that seeks to achieve objectives that reflect a common vision [1, 2].

There are few studies in our field in the perception of their leadership style of managers. No pure style, both of them are mixed but the more increase of one or the other, can either to encourage or to introduce changes and improvements.

In the primary health care (PHC) setting, managers’ leadership style is defined as a set of attitudes, behaviours, beliefs and values. This is undoubtedly influenced by the task, values, and context of management, in addition to the evolution of the health system itself [3]: most health institutions of the country still have hospital-centred values and a hierarchical culture in which economic incentives depend on best performance (*pay for performance*). Thus, a discussion about the need for transforming leaders in the clinical setting is required [4, 5].

Factors related to leadership style are divided between those focused on the leader and those focused on the context in which the leader performs. For example, leadership can be considered as simply an attribute of a person’s innate characteristics and his/her position in the hierarchy, or else it can be thought as a function distributed amongst various professionals in a group [6]. Self-perception of leadership style can also be determined by a person’s values, job satisfaction, gender, profession and management experience [7]. However, effective leadership is defined as that which tends to cause innovations [8, 9] and deep changes within the organization [10, 11]. In the context of the APS, the complexity of the processes and structures of territorial management has been increasing since the reform of primary care began in 1985 and was a major change in the organization of the whole health system [12]. In parallel, professionals and managers have developed new tasks and management functions, increasing the number of services, structures and welfare projects. Clinical leaders and managers have been distributed throughout the territory with some variability profiling, which has contributed to framing trends in management models, self-management and teamwork [13].

Scientific literature identifies and evaluates various leadership styles [14]. Traditionally, the most frequently studied form has been transactional leadership (TRL), although in recent years, transformational leadership (TFL) has been the style more frequently encountered in the literature, since it comprises the expectations and objectives of professionals, not only in their exchanges, but also in their expectations for changing values, attitudes and beliefs within the group or team [15]. Indeed, this is precisely the style that we think should be introduced in organizations that base their healthcare models on interdisciplinary and teamwork, focusing on people’s needs. Professionals with clinical experience and a comprehensive knowledge of PHC are the very factors that may ensure TFL, while carrying out all those services necessary to meet the needs and new health situations of the population [14]. While there is no clear consensus on the skills and behaviours needed to take on TFL, they include the following: competency, self-confidence, creativity, collaboration, empathy, and the ability to enjoy success. Various studies show that this style encourages teamwork and the acceptance and use of innovation by organizations, resulting in continuously improving results. That is why nowadays, PHC managers with a TFL profile area model of effective leaders prepared for change [16].

Recent studies suggest that effective leadership translates into positive results for the health system as a whole, but especially for professionals, and has a direct beneficial impact on patients [17]. Certain leadership styles have been associated with a better quality of life, as perceived by health professionals, and retention of excellent professionals increases [18]. Effective leadership strengthens professional autonomy and clinical management, allowing changes in managerial actions so they are more focused on the needs of patients and communities, and less directed towards responding to the needs of obsolete hierarchical–administrative systems.

The hypothesis of the study aims to find out if this transactional leadership style is the most self-perceived by managers in the field of Primary Health Care and if some factors such as age, sex, profession, office, managerial experience and increase are the entity providing transformational leadership.

Thus, the objectives of this study were: **(I)** to describe and learn about the self-perception of behaviours and leadership styles among PHC managers; **(II)** to determine the influence of the leadership style on job satisfaction, efficiency, effort, and willingness to work in a team; and **(III)** to determine the relationship between age, gender, profession, type of manager (Team Manager or Deputy Manager), years of management experience, and the type of organization.

## Methods

### Study design

Cross-sectional observational study.

### Study scope

The study was conducted among the 133 primary care teams in the Barcelona Health Area, This area attends populations about 1.173286 habitants with a total of 258 managers, who command multidisciplinary teams of doctors, nurses, social work professionals and administrates.

Eighty-five percent (85 %) of these teams work for the *Institut Català de la Salut* (ICS)*,* which is a public organization where professionals are employed as public servants. Other teams also participated in the study, namely Associative Entities (AE). These are professional healthcare societies that follow the entity-relationship model set down by Spanish Decree 309/97, which specifies the requirements for the accreditation of associative entities for the management of healthcare centres.

### Target population and sample size

The population eligible for the study were professionals who were Team Managers (who administer and coordinate the team) or Deputy Managers (who give support to the Team Managers). The sample included all the eligible population (*N* = 258).

Catalonian health system has management teams, consisting of doctors and nurses in the positions of Team Manager and Deputy Manager. Potential study participants were identified using the management electronic database for the Barcelona Health Area that stores information about the total number of professionals and managers identified, classified by category, title and workplace. The selection was carried out by members of the human resources unit and the Area or Sector Manager (executive director of the Barcelona Health Region).

### Study variables

#### Primary variable

The primary study variable was behavioural and leadership styles. The Multifactor Leadership Questionnaire (MLQ) developed by Bass and Avolio [19] and validated in Spanish by Vega Villa and Zavala Villalón [20] was used for this purpose. It is a multidimensional instrument that assesses how managers perceive themselves with regard to a leadership style.

This study uses the abovementioned Spanish validated version of leadership self-perception, as it is the more applicate questionnaire in the Primary Health scope showing an accurate and tested reliability.

It is a self-administered questionnaire consisting of 82 items which assess the presence or absence of various leadership styles using a scale with five points for each item (0 = never; 1 = rarely; 2 = sometimes, 3 = often; 4 = frequently) [21].

On the basis of the MLQ factorial structure, the items are grouped into three first order variables (transformational, transactional and laissez-faire) and 10 s order variables, which discriminate the different leader behaviours:

- Transformational leadership (TFL) comprises five factors: Charisma/Inspirational, Inspirational motivation, Intellectual motivation, Individualized consideration, and Behavioural and/or Attributed influence. (39 items)
- Transactional leadership (TRL) comprises three factors: Contingent reward, Passive management by exception, and Active management by exception (18 items)
- Corrective avoidant leadership (CAL) comprises two factors: Laissez-faire and Passive management by exception. (8 items)

In addition, the questionnaire includes variables which evaluate the organizational outcomes associated leadership styles: Extra effort (achievement motivation), Efficiency (ability to lead) and Satisfaction (good work atmosphere) (17 items).

### Secondary variables

The secondary variables were profession, age, gender, management experience, and type of organization.

### Data collection

Before the study got started, we asked the questionnaire’s authors for their permission to use it in our study. During June 2013, a personalized letter was sent to each of the 133 teams explaining the aim of the study and the importance of their participation to obtain a specific number of answers to validate the study. The letter also indicated that data confidentiality would be guaranteed and described the way this data would be used, highlighting the impossibility of identifying participants once the results were obtained.

A second letter was sent by e-mail once each manager gave his or her agreement to participate in the study. The e-mail included a personalized letter, signed by the principal investigator, and the questionnaire for data collection (MLQ and the fields required for the collection of secondary endpoints) was attached. The questionnaire was sent by e-mail and previously we had a pilot study with 10 managers to prevent possible mistakes.

In order to achieve an acceptable response rate of at least 60 %, 7–10 days after sending the documentation for the first time, an online postcard designed *ad hoc* was also sent thanking all managers for their participation and reminding them of the importance of their response in obtaining valid results for the study. Two or three weeks later, another letter was sent with special emphasis on the importance of their participation [22]. Data collection was performed between June and September 2013.

### Data analysis

The database was reviewed, missing or abnormal values (out of range) were explored and valid information from the data obtained from the questionnaires was retrieved. To describe quantitative data, mean and standard deviation were used if data distribution was normal; otherwise, the median and interquartile range were used. Categorical results were analysed using percentages and absolute frequencies. Estimates were reported with 95 % confidence intervals. To determine the association between leadership behaviour and styles with their positive outcomes, as well as with individual characteristics of the participants, the Chi-square test was used for categorical variables and Student’s *T*-test, variance analysis or its nonparametric equivalent were used for quantitative variables.

The correlations (Pearson’s correlation coefficients: r) between first-order and second-order variables, as well as between organizational outcomes (satisfaction, efficiency and extra effort) and leadership styles.

Logistic regression models were constructed to determine the relationship of the collected variables, using the managerial position as the dependent variable. Variable selection criteria for the estimation of models used a forward stepwise technique with an input of *p* < 0.05 and an output of *p* > 0.10.

Cronbach’s alpha was used to describe the reliability of the questionnaire and to observe the liability.

The degree of statistical significance accepted for all tests was *p* ≤ 0.05. All statistical analyses were performed using the SPSS 17.0 software for Windows.

### Ethical aspects

Data confidentiality was ensured by the anonymity of the responses. To obtain and use the data needed for sample selection, a confidentiality agreement was signed. Participants were informed in a letter from the Manager of the Health Region and a letter from the principal investigator about the study, its voluntary nature and data confidentiality. The study followed international recommendations on ethics and biomedical research in human beings, published in *International Ethical Guidelines for Biomedical Research Involving Human Subjects* of the *Council for International Organizations of Medical Sciences (CIOMS)* [23] and the Good Clinical Practice in Research Guidelines of Barcelona University (BU) [24].

To develop the study, it was obtained the approval of Ethics Committee of the clinical research at the Institute of Primary Care Research (IDIAP) Jordi Gol.

It has been respected the ethic criteria defined for the Academic Commission of the Doctoral Studies in Nursing Science at the University of Barcelona (UB).

## Results

A total of 140 responses were obtained, which means a response rate of 54.26 %. Eighty-five percent (85 %) of the surveyed managers were PHC professionals of the ICS and the remaining 15 % belonged to Associative Entities (Ref Decree) or health consortia. Most participants were nurses (61.4 %) and the predominant gender was female (75 %). Regarding work category, Team Managers were mainly physicians (71 %) and females (61.9 %). The average age of the entire sample was 49 years; the average age of the Deputy Managers was 50.5 while that of Team Managers was 47.5. With regard to the period of occupying leadership roles, most participants had been in their respective position for more than 5 years. Characteristics of respondents are shown in Table 1.

**Table 1:** Characteristics of the survey respondents (*n* = 140)

Variable	Categories	Deputy Managers (*n* = 77)	Team Managers (*n* = 63)	*p*
		*n* (%)	*n* (%)	
Profession	Physician	9 (11.69)	45 (71,43)	0.001
	Nurse	68 (88.31)	18 (28,57)	
Gender	Male	11 (14.29)	24 (38,10)	0.001
	Female	66 (85.71)	39 (61,90)	
Organization	ICS	70 (90.91)	49 (77,78)	0.03
	non ICS	7 (9.09)	14 (22,22)	
Years of management experience	<2 years	8 (10.39)	4 (6,35)	0.692
	2–5 years	17 (22.08)	14 (22,22)	
	>5 years	52 (67.53)	45 (58,44)	
Age (in years)	Mean (SD)	50.8 (7,4)	47.5 (7,3)	0.012

*ICS* Institut Català de la Salut, *SD* Standard Deviation

The results obtained by applying the Cronbach’s alpha coefficient show that the instrument used was highly reliable (α = 0.924) on all 82 items, proving its homogeneity.

### First-order and second-order variables (Table 2)

**Table 2:** Leadership styles in relation to position, profession and gender

	Position		Profession		Gender	
	Deputy	Manager	Nurse	Physician	Male	Female
TFL	3.31 (0.33)	3.3 (0.3)	3.34 (0.32)	3.24 (0.31)	3.24 (0.31)	3.32 (0.32)
Charisma/Inspirational	3.32 (0.34)	3.32 (0.29)	3.36 (0.32)	3.27 (0.31)	3.26 (0.33)	3.34 (0.31)
Inspirational motivation	3.31 (0.39)	3.28 (0.37)	3.35 (0.38)	3.22 (0.38)	3.21 (0.37)	3.33 (0.38)
Intellectual motivation	3.26 (0.39)	3.23 (0.38)	3.3 (0.39)	3.17 (0.37)	3.2 (0.38)	3.26 (0.39)
Individualized consideration	3.26 (0.39)	3.18 (0.35)	3.31 (0.36)	3.09 (0.37)	3.13 (0.35)	3.26 (0.38)
Behavioural influence	3.47 (0.38)	3.53 (0.32)	3.5 (0.38)	3.49 (0.32)	3.47 (0.36)	3.5 (0.36)
Attributed influence	3.17 (0.36)	3.12 (0.35)	3.21 (0.35)	3.06 (0.35)	3.07 (0.37)	3.18 (0.34)
TRL	3.34 (0.38)	3.28 (0.32)	3.38 (0.35)	3.2 (0.34)	3.18 (0.36)	3.35 (0.34)
Contingent reward	3.43 (0.45)	3.41 (0.35)	3.48 (0.42)	3.34 (0.37)	3.26 (0.44)	3.48 (0.38)
Passive management by exception	1.06 (0.63)	1.05 (0.49)	1.03 (0.6)	1.08 (0.53)	1.24 (0.53)	0.99 (0.58)
Active management by exception	3.05 (0.51)	3.03 (0.47)	3.07 (0.5)	2.99 (0.48)	2.91 (0.5)	3.08 (0.49)
CAL	1.82 (0.42)	1.73 (0.27)	1.79 (0.41)	1.76 (0.28)	1.81 (0.32)	1.77 (0.38)
Laissez faire	1.47 (0.56)	1.26 (0.42)	1.4 (0.54)	1.33 (0.47)	1.4 (0.54)	1.37 (0.51)
Passive management by exception	1.29 (0.57)	1.17 (0.39)	1.24 (0.54)	1.23 (0.43)	1.33 (0.48)	1.21 (0.5)
Positive/organizational outcomes						
Satisfaction	3.05 (0.4)	2.98 (0.36)	3.08 (0.38)	2.91 (0.38)	2.96 (0.35)	3.04 (0.39)
Efficiency	3.02 (0.4)	3.02 (0.37)	3.03 (0.39)	3 (0.38)	2.91 (0.37)	3.05 (0.38)
Extra effort	3.03 (0.43)	2.95 (0.39)	3.04 (0.41)	2.91 (0.4)	2.91 (0.39)	3.02 (0.42)

Values are the Mean (SD) obtained from a Likert-type scale with five points for each item (0 = never; 1 = rarely; 2 = sometimes, 3 = often; 4 = frequently); : *p* < 0.05

*TFL* Transformational leadership, *TRL* Transactional leadership, *CAL* Corrective avoidant leadership, *SD* Standard Deviation

In general, managers see their leadership as equally transactional and transformational (mean: 3.30). TRL was the most self-scored first-order variable obtained from nurses, who reported an average of 3.38, significantly higher than that of physicians (*p* < 0.003).

Focusing on TRL dimension, there was a significant difference between men and women (mean 3.34) and women (3.32 (*p* < 0.015) with lower scores in men than in women. Among the second-order variables, relating with the transformational leadership, it was highly scored by physicians and nurses alike, with no statistically significant differences.

Items related to both TRL and TFL showed higher mean scores in nurses (3.34 and 3.38) than in physicians (3.24 and 3.20), but differences were not statistically significant.

The score for first-order TFL variable was 3.34 for nurses and 3.24 for physicians; all second-order dimensions (Charisma/Inspirational, Inspirational motivation, Intellectual motivation, Individualized consideration, and Behavioural and/or Attributed influence.) were scored higher in nurses than physicians, with significant differences in individualized consideration (*p* < 0.001) and behavioural influence (*p* < 0.021).

Women scored higher in all items of TFL (3,32), but differences were not statistically significant.

There were no significant differences between Team Managers and Deputy Managers, except in self-rated TRL (deputies scored slightly higher than managers).

Second-order variables, laissez-faire and passive management by exception, within the first-order variable corrective/avoidance leadership were the least scored, with a significant difference in passive management by exception in men (*p* < 0.024).

Significant differences between variables and years in the position or between variables and the type of organization were not observed although all scores are higher in the ICS teams.

TFL showed a high correlation with inspirational charisma (*r* = 0.977) and TRL with individualized consideration (*r* = 0.931) Table 3.

**Table 3:** Pearson’s correlation between first and second order variables, as well as between organizational outcomes and leadership styles

	TFL	TRL	CAL	PAL	CI	Satisf.
TFL						
TRL	0.813					
CAL	-0.017	0.068				
PAL	-0.266	-0.180	0.915			
CI	0.977	0.784	-0.025	-0.263		
Satisf.	0.646	0.666	0.09	-0.081	0.651	
Efficiency	0.724	0.710	0.057	-0.157	0.711	0.688
Extra effort	0.668	0.626	0.077	-0.137	0.679	0.584
Intellectual motivation	0.861	0.724	0.003	-0.222	0.731	0.506
Behavioural influence	0.855	0.608	-0.096	-0.321	0.868	0.442
Inspirational motivation	0.863	0.787	0.013	-0.192	0.881	0.646
Attributed influence	0.846	0.652	0.022	-0.173	0.879	0.625
Contingent reward	0.715	0.894	0.07	-0.174	0.697	0.541
Individualized consideration	0.766	0.931	0.055	-0.157	0.732	0.663
Passive management by exception	-0.276	-0.216	0.801	0.905	-0.274	-0.074
Active management by exception	0.586	0.592	0.307	-0.103	0.560	0.413
Laissez faire	-0.220	-0.124	0.878	0.935	-0.216	-0.075

Both TRL and TFL correlated highly with efficiency (*r* = 0.724 and *r* = 0.710 respectively). On the other hand, satisfaction showed mean correlations with almost all factors except corrective/avoidance leadership.

A logistic regression model was constructed to study the relationship between the position (Team Manager or Deputy Manager) and the various independent variables: years in the position, gender, profession (nurse or physician), organization (ICS or non ICS) and all the leadership styles.

As for the influence of all study variables on the probability of being a Team Manager or Deputy Manager, the only variable with a significant statistical relationship was the profession of physician. The odds ratio (OR) of a physician being Deputy Manager was 0.04 (95 % CI: 0.01–0.12; *p* < 0.001), thus a relationship between being a physician and having a managerial position is observed.

## Discussion

The factorial structure of the MLQ obtained in this study is partly similar to that presented by Bass and Avolio [18], in which a clear distinction was found between TFL and TRL styles, especially laissez-faire. However, as already confirmed by the authors of the instrument and the numerous authors who have used the MLQ in their studies [24], small differences can be identified that may be due to cultural differences, environment and employment context.

It should be noted that the MLQ questionnaire has been used mainly in the format that assesses managers from the point of view of other professionals. However, in this study, we aimed to evaluate the self-perception that managers have of their own leadership style, which may also explain differences with other authors.

Study results have some similarities with those obtained by Morales and Molero, who also observed a predominance of TRL compared to TRL, although in their case, the MLQ was completed by professionals and not by managers [25]. Likewise, Ana Rodríguez Gonzalo studied leadership in the hospital setting, using the MLQ, and identified both styles with a clear predominance of TRL [26].

In general, most managers, regardless of their gender, give themselves high scores in the socially desirable factors (TFL and TRL) and low scores on the least valued factors (e.g. laissez-faire).

This tendency to self-praise is also confirmed by observing the perceptions of efficiency, extra effort and satisfaction expressed by managers, especially when they are nurses.

With regard to the main objective of the study (to determine self-perceived leadership style and behaviour of PHC managers), we can state that, taken as a whole, managers see themselves to having a transactional style; this observation is clearly significant in female nurse managers. It is worth mentioning that in the “contingent reward” factor, female managers also give themselves higher scores than men, which may reflect a greater tendency towards maintaining the status quo of the team.

The “contingent reward” factor involves using psychological rewards, such as acknowledgement of a job well done. Since female managers are more likely oriented to the development of their professionals and to taking care of their personal and professional growth, this could explain their greater acknowledgement of the teams they lead, and consequently, their higher self-assessment in this domain than in men.

On the other hand and as mentioned above, leaders’ self-assessment probably reflects not only their own behaviour, but also the stereotype that exists in our society about leadership.

It should also be mentioned that, in general, nurse managers score themselves as significantly more transformational in “individualized consideration” and “idealized influence -attributes”. If we take into account that there is a greater number of female nurses, this result shows great similarities with those obtained by Molero and Isabel Cuadrado [27], in which women in general were more transformational, although not significantly.

In relation to gender, we noted that it has been obtained the majority of the answers by women and it is also noted that women perceive themselves significantly more transactional than men (3.35 vs 3.18), as well as more transformational, although differences in the score are smaller (3.32 and 3.24, respectively). These results are in line with those obtained by authors such as Bass and Avolio [28], Bass and cols. [29], but they are contradicted by other studies, such as the one conducted by Druskat [30].

Finally, with respect to the laissez-faire factor, like Bass [31], we found that women tend to have less passive leadership behaviours than men, although differences are on the verge of significance (1.24 vs. 0.99).

Variables such as age, type of organization, and years in the position do not have too much effect on leadership styles in general. Only in the Catalonian Health Institute teams did we see higher scores in both transactional and transformational styles.

Regarding correlations, the study also found high correlations between TFL and satisfaction and efficiency scores. In general, correlations between TFL and organizational factors, they are high, coinciding with the investigations of Bass and Avolio [19] and many more studies. There is, however, an important difference between men and women and being a physician or a nurse: for nurses, the TFL is more related to subordinate efficiency rather than satisfaction, while the opposite happens in the case of physicians. A possible interpretation of this result would be that since fewer nurses are traditionally Team Managers than physicians, they think that a good leadership style (transformational) increases efficiency, resulting in satisfaction of professionals.

The opposite happens with physicians, since they take for granted their team efficiency and consider that TFL increases satisfaction.

In general, we can see a management culture that is less transformational than we thought, although both styles are present in the managers’ self-perception. There is still a direct relationship between Team manager and physician (*p* < 0.001) and the Deputy Manager is usually a nurse. Therefore, profession could determine a person’s access to the position of manager. In this regard, determining the reasons for the access of fewer nurses to the position of Team Manager and how to favour equal opportunities, especially if management-training’s opportunities are equal or similar, are factors that need further study.

More studies in which the views of professionals regarding the leadership styles of their managers are required to answer how they perceive their leaders and the impact these leaders have on their daily lives.

## Study limitations

The main limitation of the study consisted of a relatively low response rate to the self-administered questionnaires sent by e-mail. Strategies implemented to improve this rate were: prior notification, personalized letters, prepaid reply envelopes [20, 21, 32].

However, the majority of the questionnaires have been completed totally, even its high number of items, so the possibility of an error “no answer” it is reduced. Nevertheless, in the questionnaire, has been participated professionals with many different management profiles.

Although we cannot know the characteristics of the people who did not answered or did not answer correctly, given the anonymity of the survey, the similarities between the socio-demographic characteristics of study subjects and those of the expected population of primary care managers in our setting allow us to be reasonably confident of the validity of the results obtained.

The vast majority of the responses to items included in the MLQ questionnaire may have been strongly influenced by the principle of social desirability (as it was expected), although this impact should be reduced by the anonymous nature of the questionnaire. The length of the questionnaire may condition the response rate (response exhaustion, lack of time) [33].

As the teams included belong only to the Barcelona area, this implies that the results are less generalizable.

## Conclusions

Multifactor Leadership Questionnaire (MLQ) is one of the most widely used tools to measure leadership, as people perceive it. The questionnaire is supported by a solid theoretical and empirical basis and by its extensive application, both internationally and nationally, in measuring leadership styles and behaviours.

Describing how PHC managers (Team and Deputy Managers) perceive their own leadership style offers us updated information in the primary health care setting. The results have exposed a scenario in which two leadership styles coexist, although TRL seems to be the predominant one, as discussed in the study hypothesis. This could be due to the traditional management strategy to praise and economically reward, based on instruments such as management by objectives, established more than 10 years ago in the majority of organizations of our health care model.

If we take into account that the transformational styles are characterized as generators of self-confidence, they should match the perception of support, guidance and training by the health care professionals. Therefore, more studies exploring the vision of professionals should be conducted.

In today’s dynamic environment in which populations are constantly changing, transformational leaders would represent a key factor for better achievement of goals and health outcomes.

## Abbreviations

AE: Associative entities
CAL: Corrective avoidant leadership
CI: Charisma/Inspirational
ICS: Institut Català de la Salut
MLQ: Multifactor Leadership Questionnaire
OR: Odds ratio
PAL: Passive avoidant leadership
PHC: Primary health care
Satisf.: Satisfaction
SD: Standard deviation
TFL: Transformational leadership
TRL: Transactional leadership
